# Relationship between Vitreous Levels of Matrix Metalloproteinases and Vascular Endothelial Growth Factor in Proliferative Diabetic Retinopathy

**DOI:** 10.1371/journal.pone.0085857

**Published:** 2013-12-31

**Authors:** Ahmed M. Abu El-Asrar, Ghulam Mohammad, Mohd. Imtiaz Nawaz, Mohammad Mairaj Siddiquei, Kathleen Van den Eynde, Ahmed Mousa, Gert De Hertogh, Ghislain Opdenakker

**Affiliations:** 1 Department of Ophthalmology, College of Medicine, King Saud University, Riyadh, Saudi Arabia; 2 Laboratory of Histochemistry and Cytochemistry, University of Leuven, KU Leuven, Belgium; 3 Rega Institute for Medical Research, Department of Microbiology and Immunology, University of Leuven, KU Leuven, Belgium; National Center for Scientific Research Demokritos, Greece

## Abstract

To investigate which matrix metalloproteinases (MMPs) are more likely to be involved in the angiogenic process in proliferative diabetic retinopathy (PDR), we measured the levels of MMPs in the vitreous fluid from patients with PDR and controls and correlated these levels with the levels of vascular endothelial growth factor (VEGF). Vitreous samples from 32 PDR and 24 nondiabetic patients were studied by mosaic multiplex MMPs enzyme-linked immunosorbent assay (ELISA), single ELISA, Western blot and zymography analysis. Epiretinal membranes from 11 patients with PDR were studied by immunohistochemistry. MMP-8 and MMP-13 were not detected. ELISA, Western blot and gelatin ymography assays revealed significant increases in the expression levels of MMP-1, MMP-7, MMP-9 and VEGF in vitreous samples from PDR patients compared to nondiabetic controls, whereas MMP-2 and MMP-3 were not upregulated in vitreous samples from PDR patients. Significant correlations existed between ELISA and zymography assays for the quantitation of MMP-2 (r=0.407; p=0.039) and MMP-9 (r=0.711; p<0.001). Significant correlations were observed between levels of VEGF and levels of MMP-1 (r=0.845; P<0.001) and MMP-9 (r=0.775; p<0.001), and between levels of MMP-1 and MMP-9 (r=0.857; p<0.001). In epiretinal membranes, cytoplasmic immunoreactivity for MMP-9 was present in vascular endothelial cells and stromal monocytes/macrophages and neutrophils. Our findings suggest that among the MMPs measured, MMP-1 and MMP-9 may contribute to the angiogenic switch in PDR.

## Introduction

 Proliferative diabetic retinopathy (PDR), a long-term complication of diabetes, is characterized by vasculopathy associated with abnormal angiogenesis and expansion of extracellular matrix (ECM) resulting in the outgrowth of fibrovascular membranes at the vitreoretinal interface. Formation of fibrovascular tissue results in severe complications such as vitreous hemorrhage and traction retinal detachment. Angiogenesis, the sprouting of new blood vessels from preexisting blood vessels, is a multistep process requiring the degradation of the basement membranes and ECM, endothelial cell migration, endothelial cell proliferation, and capillary tube formation [1]. Vascular endothelial growth factor (VEGF) is the major angiogenic factor in PDR that promotes neovascularization and vascular leakage [2].

 The angiogenic switch involves in part the proteolytic degradation of basement membranes and ECM components by matrix metalloproteinases (MMPs). In addition to removing the physical barriers to new vessel growth, MMPs proteolytically release VEGF from the ECM-associated reservoirs [3,4], resulting in increased VEGF bioavailability and triggering the VEGF-driven angiogenic switch [3,4]. MMPs are a family of zinc ion-binding Ca^2+^-dependent neutral endopeptidases that act together or in concert with other enzymes to degrade most components of the ECM. At least 25 MMP members have been indentified and are divided into collagenases (MMP-1, MMP-8, and MMP-13), gelatinases (MMP-2, and MMP-9), stromelysins (MMP-3, MMP-10, and MMP-11), matrilysins (MMP-7, and MMP-26), membrane-type MMPs, and others [5]. Most of the MMPs are inhibited by specific endogenous tissue inhibitors which are known as tissue inhibitors of matrix metalloproteinases (TIMPs) [5].

 Under steady state physiologic conditions, the expression of MMPs in most tissues is relatively low, with the possible exception of MMP-2, which appears to be expressed constitutively [1]. These enzymes have been implicated in invasive cell behavior and recent studies have indicated that MMPs are generally up-regulated in several conditions that accompany angiogenesis and play an important role in the initiation of angiogenesis [1]. In PDR, the levels of certain MMPs are increased dramatically [6-9]. This up-regulation of MMPs is linked to angiogenesis and progression of PDR. However, the relative relevance of individual MMPs to angiogenesis associated with PDR remains to be elucidated. To develop efficient specific inhibitors for anti-angiogenic therapy, it is necessary to know which MMPs are more likely to be involved in the angiogenic process in PDR. Therefore, we measured the levels of the MMPs MMP-1, MMP-2, MMP-3, MMP-7, MMP-9, and MMP-13 in the vitreous fluid from patients with PDR and nondiabetic patients and correlated their levels with the levels of the angiogenic factor VEGF. The association of MMPs with VEGF is likely to be an indicator of the relevance of MMPs to angiogenesis and diabetic retinopathy.

## Materials and Methods

### Ethics statement

The study was conducted according to the tenets of the Declaration of Helsinki. All the patients were candidates for vitrectomy as a surgical procedure. All patients signed a preoperative informed written consent and approved the use of the excised epiretinal membranes and vitreous fluid for further analysis and clinical research. The study design and the protocol was approved by the Research Centre and Institutional Review Board of the College of Medicine, King Saud University.

The sections from the control patients were obtained from patients treated at the University Hospital, University of Leuven, Belgium, in full compliance with tenets of the Declaration of Helsinki. We used archived material and patients gave written consent at admission for the use of the leftover material in studies. The Ethics Committee of the University Hospital, University of Leuven approved this consent procedure.

### Vitreous samples and epiretinal membrane specimens

Undiluted vitreous fluid samples (0.3 - 0.6 ml) were obtained from 32 patients with PDR during pars plana vitrectomy. The indications for vitrectomy were traction retinal detachment, combined traction/rhegmatogenous retinal detachment, and/or nonclearing vitreous hemorrhage. The control group consisted of 24 patients who had undergone vitrectomy for the treatment of rhegmatogenous retinal detachment with no proliferative vitreoretinopathy. Controls were free from systemic disease. Vitreous samples were collected undiluted by manual suction into a syringe through the aspiratin line of vitrectomy, before opening the infusion line. The samples were centrifuged (5000 rpm for 10 min, 4°C) and the supernatants were aliquoted and frozen at -80°C until assay. Epiretinal fibrovascular membranes were obtained from 11 patients with PDR during pars plana vitrectomy for the repair of traction retinal detachment. Membranes were fixed in 10% formalin solution and embedded in paraffin. 

### Enzyme-linked immunosorbent assay

 Enzyme-linked immunosorbent assay (ELISA) kits for a panel of specific human MMPs panel (mosaic multiple matrix metalloproteinases, Cat No: MEA006), Human MMP-2 (Quantikine MMP-2, Cat No: DMP2F0) and human VEGF (Quantikine Human Vascular Endothelial Growth Factor, Cat No: DVE00) were purchased from R&D Systems, Minneapolis, MN. 

 The mosaic human MMPs panel immunoassay simultaneously detects seven MMPs with members of the collagenase (MMP-1, MMP-8, MMP-13) the gelatinase (MMP-2, MMP-9), the stromelysin (MMP-3) and the matrilysin (MMP-7) subgroups. The detection limit for each MMP using MMPs panel ELISA kit was 28, 120, 4, 2, 22, 45, and 36 picograms/mL (pg/mL) for MMP-1, MMP-2, MMP-3, MMP-7, MMP-8, MMP-9, and MMP-13, respectively. The detection limits for MMP-2 single ELISA kit and VEGF ELISA kit were 47 pg/mL and 5 pg/mL, respectively. The ELISA plate readings were done using FLUOstar Omega-Microplate reader from BMG Labtech, Offenburg, Germany. 

### Measurement of human MMPs

The detection and quantification of MMPs in the vitreous fluid was done using mosaic multiplex MMPs ELISA kit according to the manufacturer’s instruction. An amount of 50 µL of cocktail of recombinant human MMPs standard and undiluted vitreous fluid, respectively, were added to each well of the ELISA plate. The assay was performed in duplicate for each standard and vitreous sample. The plates were incubated and washed followed by the addition of detection antibody. After incubation and washing, the Streptavidin-HRP was added into each well. Immunoreactivity for each MMP was visualized as spot on a high-performance chemiluminescence machine (G: Box Chemi-XX8, Cambridge, UK) by the addition of enhanced chemiluminescence plus substrate and quantified by densitometric analysis using image processing and analysis in GeneTools (Syngene by Synoptic Ltd. Cambridge, UK). The 5-parameter fit logistic (5-PL) curve equation (MasterPlex EX 2010 software, Hitachi, San Francisco, CA) was used for making the standard curve and the actual concentration for each sample was calculated. 

### Measurement of human MMP-2 and VEGF

The quantification of MMP-2 and VEGF in the vitreous fluid was determined using ELISA kits according to the manufacturer’s instruction. For each ELISA kit, an undiluted standard served as the highest standard level and the calibrator diluents served as the zero standard. Depending upon the detection range for each ELISA kit, the supernatant vitreous obtained was used either directly or diluted with calibrator diluents supplied to the ELISA kit. 

For the measurement of MMP-2, 50 µL of 10-fold diluted vitreous were used and added to the respective well of ELISA plate. For the measurement of VEGF, an amount of 100 µL of undiluted vitreous fluid was added to each well of the ELISA plate. The assay was performed in duplicate for each standard and vitreous sample. Antibody against MMP-2 or VEGF (conjugated to horseradish peroxidase) was added to each well of the ELISA plate. After the addition of substrate mix solution, the reaction was completed with the addition of stop solution (2N sulfuric acid) to each well of the ELISA plate and immediately the Optical Density (OD) was read at 450 nm with the use of a microplate reader with wavelength correction of 540 nm. Using the 4-parameter fit logistic (4-PL) curve equation for making the standard curves, the actual concentration for each sample was calculated. For the diluted vitreous fluid, the correction read from the standard curve obtained using 4-PL was multiplied by the dilution factor to obtain the actual reading for each sample.

### Zymography

Gelatinolytic levels of MMP-2 and MMP-9 were estimated in the vitreous fluid by zymography technique [10]. Equal volumes (10µl) of vitreous samples were mixed with Laemmli’s sample buffer (1:1, v/v) and were electrophoresed under nonreducing conditions onto 10% SDS-PAGE gels polymerized with 1 mg/mL gelatin. The gels were washed with 2.5% Triton X-100, and overnight incubated at 37°C in substrate buffer containing 50 mM Tris-HCl, pH 8.0, 5 mM CaCl_2_, and 0.02% NaN_3_. The gels were stained with Coomassie blue stain (0.5% Coomassie blue R-250, 5% methanol, and 10% acetic acid), followed by destaining (5% methanol, 10% acetic acid). The image was taken with the GeneSys (version 1.2.0.0) software on a G: Box machine and signal intensities of bands (for MMP-9; ~100 kDa and for MMP-2; ~70 kDa) were quantified using the GeneTools software. 

### Western blot analysis

To determine the expression of MMP-1, MMP-2, MMP-3 and MMP-9 in the vitreous samples, equal volumes of vitreous samples were boiled in Laemmli’s sample buffer (1:1, v/v) under reducing conditions for 10 min. Equal volume of lysis solution (15 µL) was loaded and separated on 10-15% SDS-PAGE gels and transferred onto nitrocellulose membranes. After protein transfer, the membrane was blocked (1.5 h, room temperature) with 5% non-fat milk and incubated overnight at 4°C with rabbit polyclonal anti-MMP-1 (1:300; Cat No: sc-30069, Santa Cruz, CA, USA), mouse monoclonal anti-MMP-2 (1:300; Cat No: ab3158, Abcam, UK) and goat polyclonal anti-MMP-3 ((1:300; Cat No: sc-30069, Santa Cruz), mouse monoclonal anti-MMP-9 (1:1000; REGA-2D9, Rega Institute for Medical Research, University of Leuven, Belgium)[11]. After incubation with primary antibody, the membranes were washed and incubated at room temperature for 1.5 h with their respective secondary horseradish peroxidase-conjugated antibody. Membranes were again washed four times and the immunoreactivity of bands was visualized on a high-performance chemiluminescence machine (G: Box) by using enhanced chemiluminescence plus Luminol (1:1 v/v, Cat No: sc-2048, Santa Cruz). Protein bands were quantified by densitometric analysis using image processing and analysis in GeneTools software.

### Immunohistochemical staining

Endogenous peroxidase in tissue sections was abolished with 2% hydrogen peroxide in methanol for 20 minutes, and nonspecific background staining was blocked by incubating the sections for 5 minutes in normal swine serum. Antigen retrieval was performed by boiling the sections in 10 mM citrate buffer [pH 6] for 30 minutes. Subsequently, the sections were incubated with mouse monoclonal anti-MMP-9 (1:50; REGA-2D9) and mouse monoclonal anti-CD45 (1:50; Dako, Glostrup, Denmark) for 60 minutes. Optimal working concentration and incubation time for the antibodies were determined earlier in pilot experiments. The sections were then incubated for 30 minutes with immunoglobulin conjugated to peroxidase-labeled dextran polymer [EnVision (Flex); Dako, Carpinteria, CA, USA]. The reaction product was visualized by incubation for 10 minutes in 0.05 M acetate buffer at pH 4.9, containing 0.05% 3-amino-9-ethylcarbazole (Sigma-Aldrich, Bornem, Belgium) and 0.01% hydrogen peroxide, resulting in bright-red immunoreactive sites. The slides were then faintly counterstained with Harris hematoxylin. To identify the phenotype of cells expressing MMP-9, sequential double immunohistochemistry was performed. Antigen retrieval was performed by boiling the sections in Tris-ethylene-diaminetetraacetic acid buffer [pH 9] for 20 minutes, followed by incubation with anti-CD45 and subsequently treated with peroxidase conjugated secondary antibody. The sections were visualized with 3,3’-diaminobenzidine tetrahydrochloride. Incubation of the second primary antibody anti-MMP-9, was followed by treatment with alkaline phosphatase conjugated secondary antibody. The sections were visualized with fast red. No counterstain was applied. Omission or substitution of the primary antibody with an irrelevant antibody from the same species and staining with chromogen alone were used as negative controls. Sections from patients with glioblastoma were used as positive controls for the immunohistochemical staining methods. 

### Statistical analysis

Data are presented as the mean ± standard deviation. The non-parametric Mann-Whitney U test was used to compare means from two independent groups. Pearson correlation coefficients were computed to investigate correlations between variables. A p-value less than 0.05 indicated statistical significance. SPSS version 19.0 (IBM Inc., Chicago, IL) and program 3S from the BMDP 2007 Statistical Package were used for the statistical analyses.

## Results

### Mosaic multiplex ELISA levels of MMPs in vitreous samples

**Figure 1:**
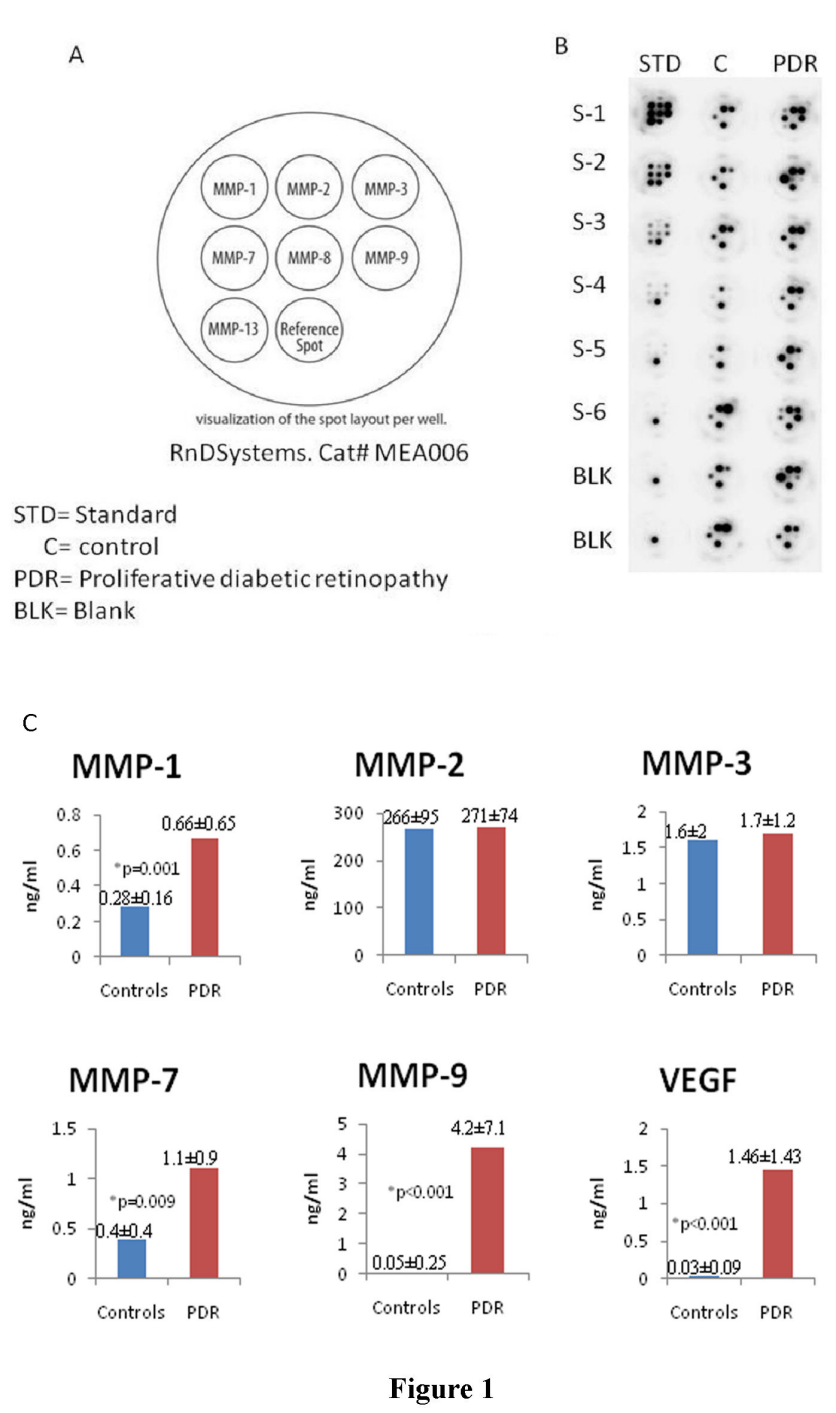
**A.** Well map indicating the spot locations of the analytes detected using the mosaic ELISA Human MMP Panel. The reference spot provides a strong positive signal for easy visualization of the well locations and spot alignment during data Analysis. **B.** Representative images of individual wells for the standard curve (left lane) and series of eight samples from nondiabetic control patients (C) and eight proliferative diabetic retinopathy (PDR) patients. **C.** Comparison of mean mosaic multiplex metalloproteinase (MMPs) enzyme-linked immunosorbent assay levels and vascular endothelial growth factor (VEGF) levels between proliferative diabetic retinopathy (PDR) patients and nondiabetic control patients. *The difference between the two means was statistically significant at 5% level of significance.

 The levels of seven different MMPs in the vitreous samples were determined by mosaic multiplex MMPs ELISA assay (Figure 1A, B). MMP-8 and MMP-13 were not detected in vitreous samples from patients with PDR (n=16) and nondiabetic control patients (n=24). Among the MMPs examined, MMP-1, MMP-2, MMP-3, and MMP-7 were detected in all vitreous samples from patients with PDR and control patients without diabetes. MMP-9 was detected in all vitreous samples from patients with PDR and in 2 of 24 (8.3%) vitreous samples from nondiabetic patients. The mean levels of MMP-1, MMP-7 , and MMP-9 in vitreous samples from PDR patients were significantly higher than those in nondiabetic patients p=0.001; p=0.009; p<0.001, respectively; Mann-Whitney test) (Figure 1C). In contrast, levels of MMP-2 and MMP-3 did not differ significantly between nondiabetic control patients and PDR patients (Figure 1C).

With the use of a single ELISA assay for MMP-2, we confirmed that the levels of MMP-2 did not differ significantly between nondiabetic control patients (n=22) (77.89±55.53 ng/ml) and PDR patients (n=30) (81.085±43.74 ng/ml) (p=0.581; Mann-Whitney test).

**Figure 2:**
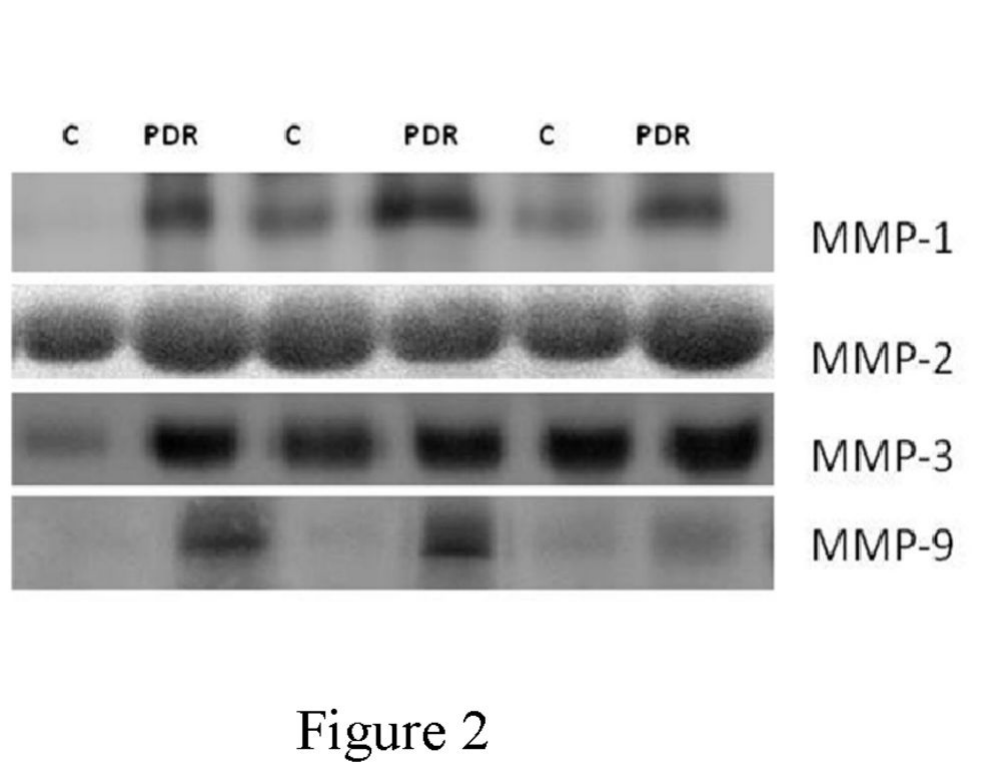
Representative Western blot analysis of matrix metalloproteinase-1 (MMP-1), MMP-2, MMP-3 and MMP-9 in vitreous samples from three proliferative diabetic retinopathy (PDR) and three nondiabetic control (C) patients.

 With the use of Western blot analysis, we confirmed that MMP-1, MMP-2, MMP-3 and MMP-9 were expressed in vitreous samples from patients with PDR and nondiabetic control patients (Figure 2).

### Zymography levels of MMP-2 and MMP-9 in vitreous samples

**Figure 3:**
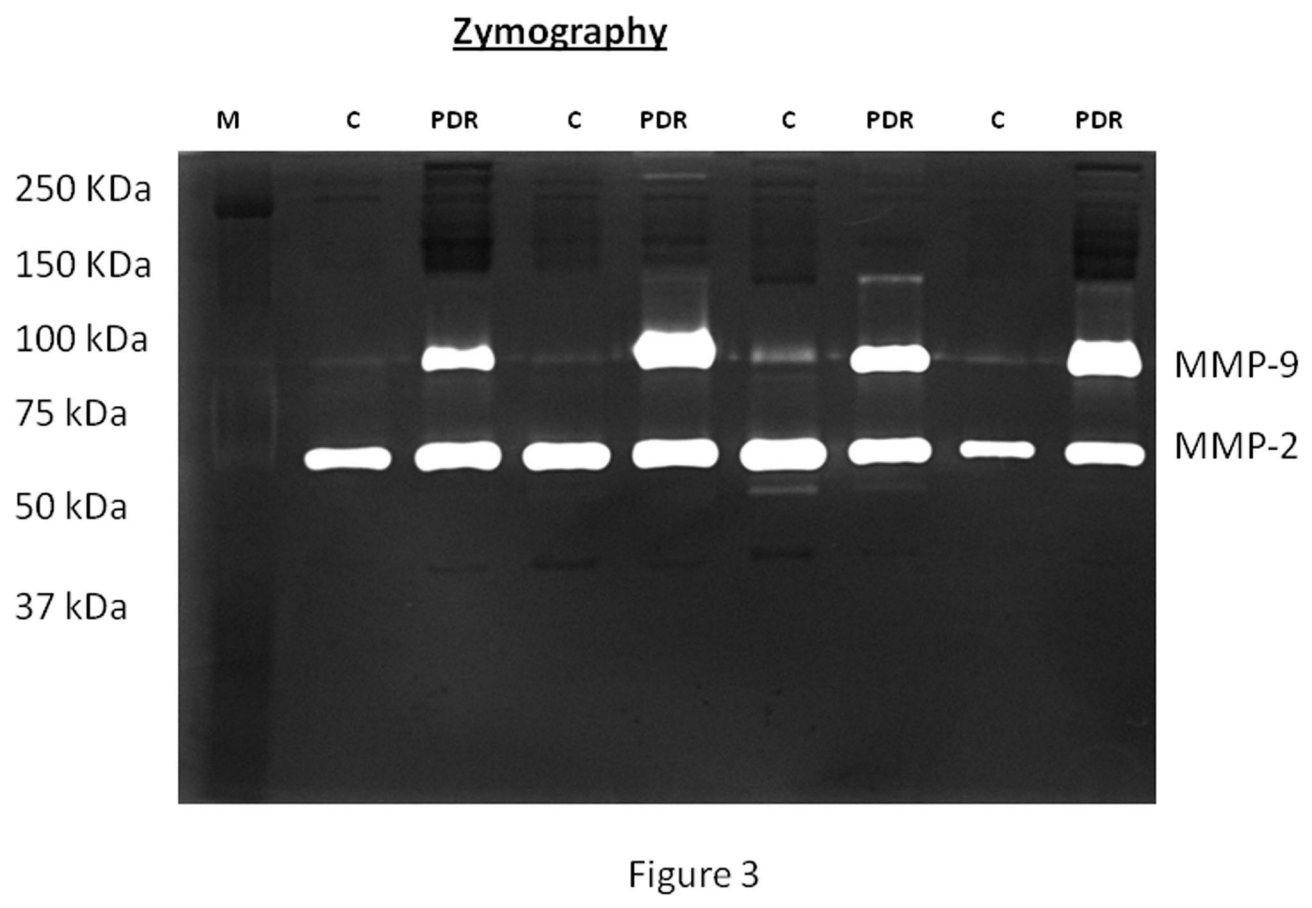
Gelatin zymography of the vitreous samples. Representative zymography of the vitreous samples from four nondiabetic control (C) patients and four patients with proliferative diabetic retinopathy (PDR) patients. Gelatinolytic bands of ~100 and ~70 kDa correspond to MMP-9 and MMP-2, respectively.

 All vitreous samples contained MMP-2 and MMP-9. The levels of MMP-2 did not differ significantly between nondiabetic control patients (n=18) (505.4±216.1 scanning units) and PDR patients (n=18) (540.9±185.6 scanning units ) (p=0.739; Mann-Whitney test). The mean MMP-9 level in patients with PDR (392.3±253.6 scanning units) was significantly higher than the mean level in nondiabetic control patients (168.2±65.0 scanning units) (p=0.011; Mann-Whitney test) (Figure 3).

### ELISA levels of VEGF in vitreous samples

 VEGF was detected in 28 out of 32 (8.75%) vitreous samples from patients with PDR, and in 3 out of 24 (1.25% ) samples from nondiabetic control patients. Mean VEGF level in vitreous samples from PDR patients (1.46±1.43 ng/ml) was significantly higher than the mean level in nondiabetic control patients (0.037±0.095 ng/ml) (p<0.001; Mann-Whitney test) (Figure 1C).

### Correlations

 Significant correlations were observed between mosaic multiplex MMPs ELISA and zymography assays for quantitation of MMP-2 (r=0.407; p=0.039) and MMP-9 (r=0.711; p<0.001). In addition, there was a significant correlation between single ELISA and zymography assays for the quantitation of MMP-2 (r=0.726; p<0.001). There was a significant correlation between mosaic multiplex MMPs ELISA and single ELISA for quantitation of MMP-2 (r=0.701; p<0.001).

**Figure 4:**
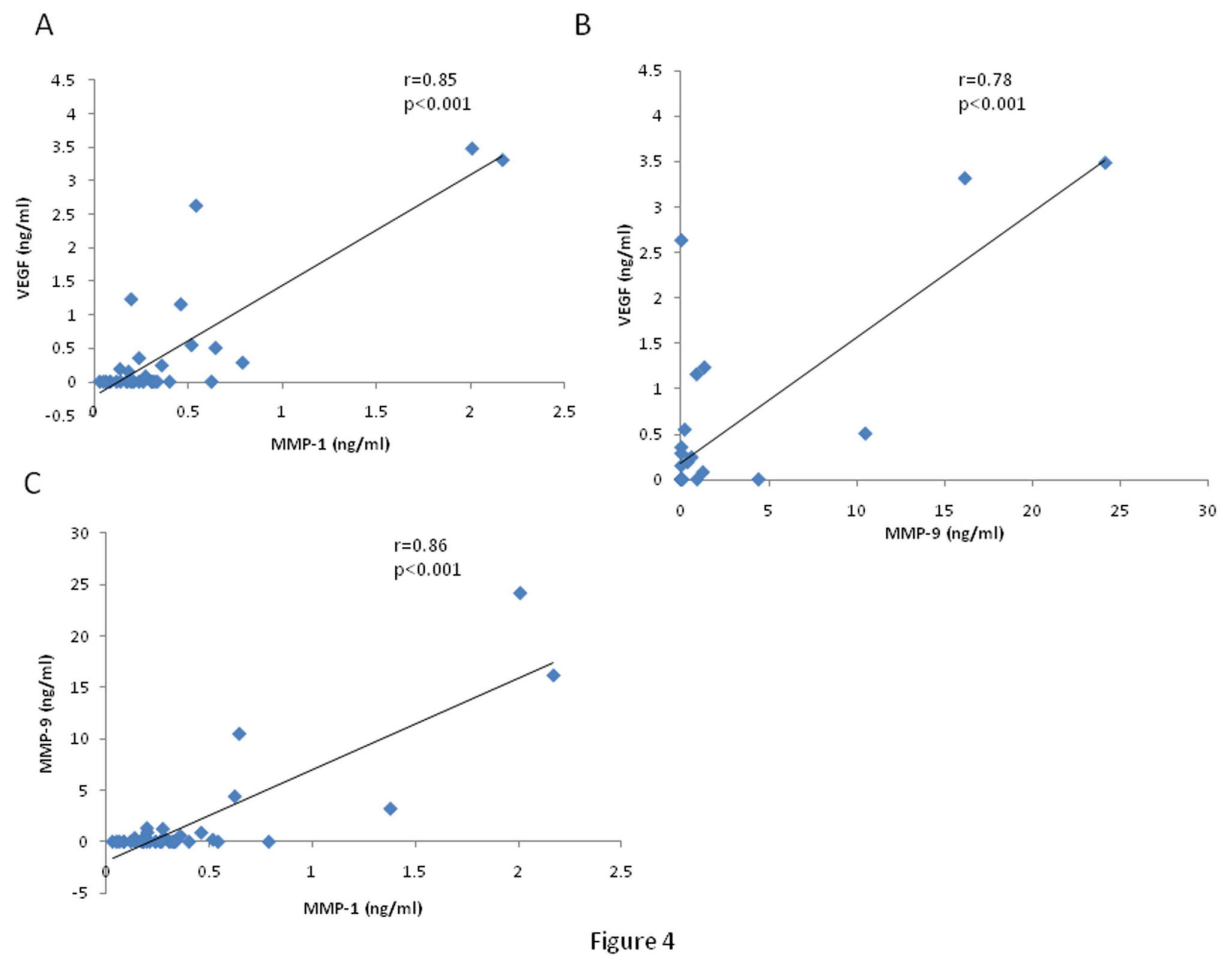
Significant positive correlations between vitreous fluid levels of vascular endothelial growth factor (VEGF) and levels of matrix metalloproteinase-1 (MMP-1) (A) and MMP-9 (B) and between vitreous fluid levels of MMP-1 and MMP-9 (C) in vitreous samples from 16 proliferative diabetic retinopathy and 24 nondiabetic control patients analyzed with mosaic multiplex matrix metalloproteinase (MMPs) enzyme-linked immunosorbent assay.

 Significant correlations were found between vitreous fluid levels of VEGF and levels of MMP-1 (r=0.845; p<0.001) and MMP-9 (r=0.775; p<0.001). There was a significant correlation between vitreous fluid levels of MMP-1 and MMP-9 (r=0.857; p<0.001) (Figure 4). In addition, MMP-9 zymography levels correlated significantly with vitreous fluid levels of MMP-1 (r=0.856; p<0.001) and VEGF (r=0.872; p<0.001).

### Immunohistochemical analysis

**Figure 5:**
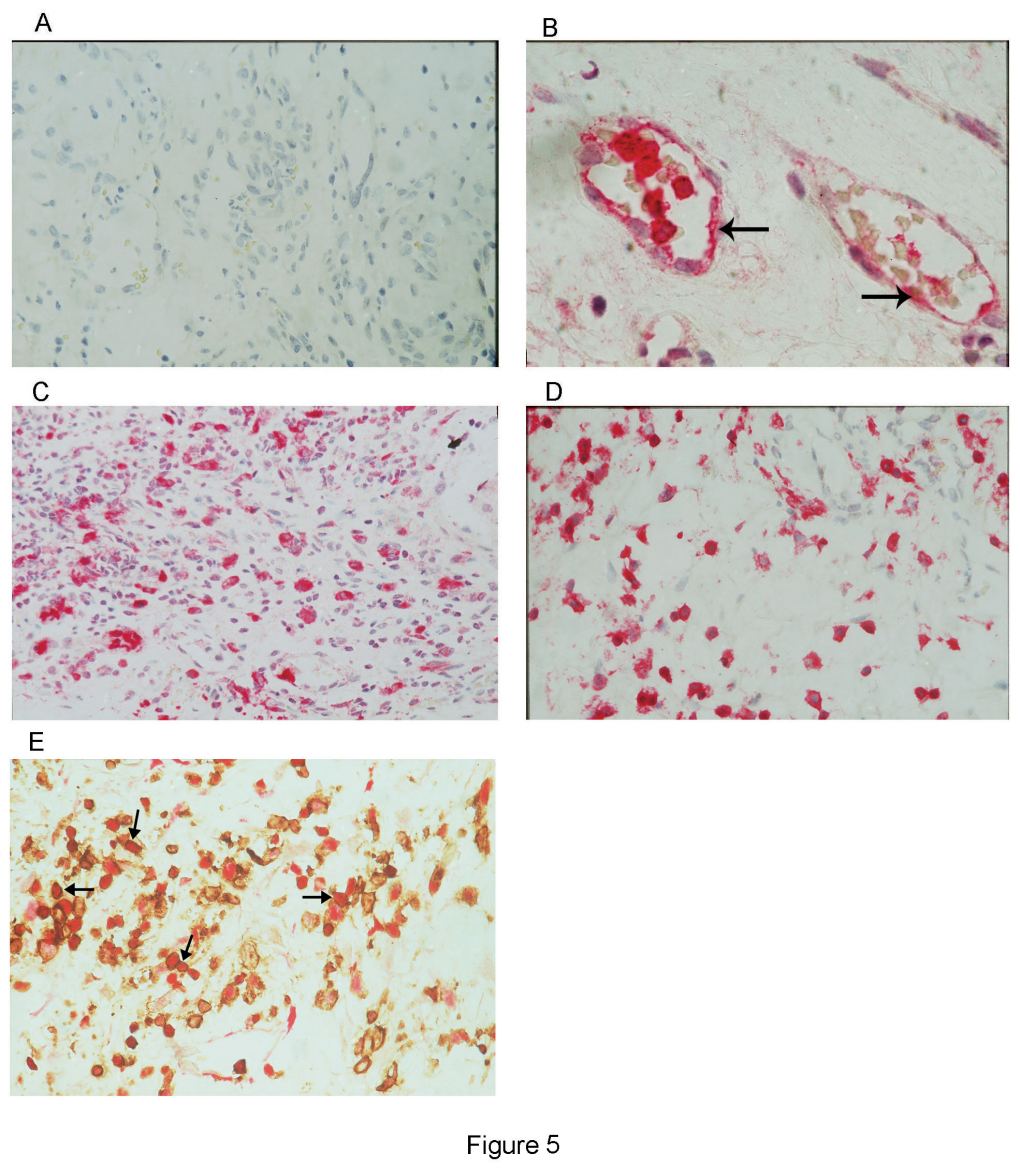
Proliferative diabetic retinopathy epiretinal membranes. Negative control slide that was treated identically with an irrelevant antibody showing no labeling (A). Immunohistochemical staining matrix metalloproteinase-9 (MMP-9) showing immunoreactivity in endothelial cells (arrows), intravascular leukocytes (B) and stromal cells (C). Immunohistochemical staining for CD45 showing immunoreactivity in leukocytes expressing the leukocyte common antigen CD45 (D) Double immunohistochemistry for CD45 (brown) and MMP-9 (red) showing cells co-expressing CD45 and MMP-9 (arrows). No counterstain was applied (E) (original magnification X40).

 No staining was observed in the negative control slides (Figure 5A). Immunoreactivity for MMP-9 was present in all membranes and was detected in the cytoplasm of stromal cells, intravascular leukocytes and vascular endothelial cells (Figure 5B, C). The majority of MMP-9-positive stromal cells were monocytes/macrophages and neutrophils. In serial sections, the distribution and morphology of stromal cells expressing MMP-9 were similar to those of cells expressing the leukocyte common antigen CD45 (Figure 5D). Double staining confirmed that stromal cells expressing MMP-9 co-expressed CD45 (Figure 5E).

## Discussion

 Aside the technological aspects by which various protein measurement methods were validated and corroborated, four key findings emerged from this study. First, of the three collagenases (MMP-1, MMP-8 and MMP-13), only MMP-1 was detected in the vitreous fluid from nondiabetic control patients and patients with PDR. Furthermore, MMP-1 was significantly upregulated in the vitreous fluid from patients with PDR. Second, of the gelatinases (MMP-2, and MMP-9), MMP-9, but not MMP-2, was significantly increased in vitreous samples from eyes with PDR compared with those of nondiabetic patients. Third, MMP-7 levels in the vitreous fluid from patients with PDR were significantly higher than those in nondiabetic patients. Fourth, there were significant positive correlations between the levels of VEGF and the levels of MMP-1 and MMP-9. Collectively, these findings indirectly suggest that in PDR, MMP-1 and MMP-9 may play an important role in the progression of angiogenesis associated with PDR.

 MMP-1 is an interstitial collagenase that is often upregulated in several types of cancer, and this enzyme is involved in tumor-induced angiogenesis [12-15]. Several studies demonstrated that enhanced MMP-1 expression increased angiogenic activity in tumors [12-15], and that tumor-derived MMP-1 stimulates endothelial cell activation and promotes complex tube formation *in vitro* [13,14]. In addition, attenuation of MMP-1 expression reduced tumor-induced angiogenesis *in vivo* [14]. Furthermore, MMP-1 acts directly on human microvessel endothelial cells as a pro-angiogenic signaling molecule and induces the expression of different subsets of pro-angiogenic genes. Purified MMP-1 is also capable of inducing angiogenesis in vivo in a controlled Matrigel system, thereby defining MMP-1 as a pro-angiogenic factor [16]. Several studies demonstrated the presence of a positive feedback regulation between MMP-1, MMP-9 and VEGF. VEGF stimulation significantly enhanced production of MMP-1 and MMP-9 by immortalized human chondrocytes [17] and human vascular smooth muscle cells [18]. MMP-1 induces MMP-9 expression in human microvessel endothelial cells [16] and macrophages [19]. In agreement with these studies, we demonstrated significant correlations between the levels of MMP-1 and the levels of MMP-9 and VEGF in the vitreous fluid. 

 In the present study, MMP-7 levels in the vitreous samples from eyes with PDR were significantly increased as compared to nondiabetic control patients. MMP-7 contributes to angiogenesis by breaking down basement membranes. MMP-7 expression was demonstrated in endothelial cells of various tumors and was associated with decreased survival suggesting that MMP-7 is involved in tumor angiogenesis [20]. Furthermore, MMP-7 has been shown to accelerate the proliferation of human umbilical vein endothelial cells in a dose-dependent manner *in vitro* suggesting that MMP-7 can directly induce angiogenesis [21]. Recently, it was demonstrated that MMP-7 degrades soluble VEGF receptor-1 resulting in the escape of VEGF from sequestration by soluble VEGF receptor-1. These findings provide a mechanism for the regulation of VEGF bioavailability within the local endothelial microenvironment [22].

 MMP-9 expression is low or absent in most normal tissues, and it is markedly elevated during inflammatory, autoimmune, degenerative and neoplastic diseases and angiogenic lesions [23]. On the other hand, expression of MMP-2 is constitutive and most proinflammatory stimuli fail to increase the expression level of MMP-2 [1,23]. In the present study, zymography analysis and ELISA assays confirmed previous studies reporting that MMP-9, but not MMP-2, is increased in the vitreous fluid from patients with PDR compared with those of nondiabetic patients [6,8,9]. However, it was reported by Noda et al. [7] that MMP-2 is also significantly increased in vitreous samples from patients with PDR. Our findings suggest that MMP-2 apparently does not significantly contribute to the angiogenic switch in PDR. Our findings are consistent with previous studies using animal models of cancer that indicated that MMP-2 apparently does not significantly contribute to the angiogenic switch [1]. Our analysis showed a significant positive correlation between the vitreous levels of VEGF and MMP-9. VEGF is a critical regulator of angiogenesis that stimulates proliferation, migration, and proteolytic activity of endothelial cells [24]. Similarly, previous studies reported a strong correlation between the expression of VEGF and MMP-9, but not with MMP-2, in cancer patients [3,25,26], and in the synovial fluid of patients with rheumatoid arthritis [27]. This correlation is consistent with preclinical data reporting the presence of a positive feedback regulation between VEGF and MMP-9. VEGF treatment can induce MMP-9, but not MMP-2 expression in different cells [18,28]. Conversely, exogenous MMP-9 increased the secretion of VEGF, whereas MMP-2 reduced the secretion of VEGF [28]. 

 Growing evidence indicates that inflammatory cells, including monocytes/macrophages and neutrophils, constitute a major cellular source of the proangiogenic MMP-9 and contribute to tumor angiogenesis [3,29,30]. Inflammatory neutrophils deliver unique TIMP-1-free proMMP-9, which in turn rapidly and potently triggers the angiogenic switch in tumors [30-32]. In the present study, we described the *in situ* localization of the expression of MMP-9 in leukocytes expressing the leukocyte common antigen CD45 and endothelial cells in epiretinal membranes from patients with PDR. Previous studies demonstrated that activated endothelial cells over-express MMP-1 and MMP-9 during sprouting and formation of lumina-containing tubules [1].

The vitreous fluid, collected from patients with PDR during pars plana vitrectomy, is an ideal material for analysis of local, intraocular concentrations of selected proteins which take part of this pathology. However, when measuring these factors in the vitreous, some considerations should be kept in mind. Vitreous hemorrhage, associated with active neovascularization or traction on the retina induced by involuted fibrovascular proliferation during posterior vitreous detachment, can provide an influx of serum proteins into vitreous fluid. In a previous study, we demonstrated that there was no correlation between hemoglobin levels, as a measure of the amount of erupted blood, and total protein levels in vitreous fluid from patients with PDR [6]. Although VEGF is abundant in serum, the levels of VEGF in the vitreous fluid did not differ significantly between eyes with massive vitreous hemorrhage without diabetic retinopathy and nondiabetic eyes without vitreous hemorrhage. On the other hand, the concentration of VEGF was significantly higher in eyes with PDR than in nondiabetic eyes with massive vitreous hemorrhage or nondiabetic eyes with no vitreous hemorrhage [33]. Moreover, in a previous study, we reported that brain-derived neurotrophic factor (BDNF) was not detected in vitreous samples from patients with PDR and nondiabetic control patients, whereas BDNF was detected in all serum samples from patients with PDR and nondiabetic controls [34]. In the present study, we demonstrated the expression of MMP-9 by vascular endothelial cells and stromal cells in PDR fibrovascular epiretinal membranes. These findings suggest that local cellular production is the relevant source of the studied factors within the ocular microenvironment and that systemic inflow mechanism is rather improbable.

 In conclusion, among all the MMPs measured, only MMP-1 and MMP-9 significantly positively correlated with VEGF. These results suggest that both MMP-1 and MMP-9 contribute to the angiogenic switch critical for PDR progression. Combination therapy of VEGF inhibitors with MMP inhibitors may be useful in the treatment of PDR and other retinal diseases with pathological angiogenesis.

## References

[1] Deryugina EI, Quigley JP (2010) Pleiotropic roles of matrix metalloproteinases in tumor angiongenesis: contrasting, overlapping and compensatory functions. Biochim Biophys Acta 1803: 103-120. doi:10.1016/j.bbamcr.2009.09.017. PubMed: 19800930. 19800930 PMC2824055

[2] Spranger J, Pfeiffer AF (2001) New concepts in pathogenesis and treatment of diabetic retinopathy. Exp Clin Endocrinol Diabetes 109 Suppl 2: S438-S450. doi:10.1055/s-2001-18601. PubMed: 11460590. 11460590

[3] Hawinkels LJ, Zuidwijk K, Verspaget HW, de Jonge-Muller ES, van Duijn W et al. (2008) VEGF release by MMP-9 mediated heparan sulphate cleavage induces colorectal cancer angiogenesis. Eur J Cancer 44: 1904-1913. doi:10.1016/j.ejca.2008.06.031. PubMed: 18691882. 18691882

[4] Ebrahem Q, Chaurasia SS, Vasanji A, Qi JH, Klenotic PA et al. (2010) Cross-talk between vascular endothelial growth factor and matrix metalloproteinases in the induction of neovascularization in vivo. Am J Pathol 176: 496-503. doi:10.2353/ajpath.2010.080642. PubMed: 19948826. 19948826 PMC2797907

[5] Hu J, Van den Steen PE, Sang QX, Opdenakker G (2007) Matrix metalloproteinase inhibitors as therapy for inflammatory and vascular diseases. Nat Rev Drug Discov 6: 480-498. doi:10.1038/nrd2308. PubMed: 17541420. 17541420

[6] Descamps FJ, Martens E, Kangave D, Struyf S, Geboes K et al. (2006) The activated form of gelatinase B/matrix metalloproteinase-9 is associated with diabetic vitreous hemorrhage. Exp Eye Res 83: 401-407. doi:10.1016/j.exer.2006.01.017. PubMed: 16643893. 16643893

[7] Noda K, Ishida S, Inoue M, Obata K, Oguchi Y et al. (2003) Production and activation of matrix metalloproteinase-2 in proliferative diabetic retinopathy. Invest Ophthalmol Vis Sci 44: 2163-2170. doi:10.1167/iovs.02-0662. PubMed: 12714657. 12714657

[8] Jin M, Kashiwagi K, Iizuka Y, Tanaka Y, Imai M et al. (2001) Matrix metalloproteinases in human diabetic and nondiabetic vitreous. Retina 21: 28-33. doi:10.1097/00006982-200102000-00005. PubMed: 11217926. 11217926

[9] Kosano H, Okano T, Katsura Y, Noritake M, Kado S et al. (1999) ProMMP-9 (92 kDa gelatinase) in vitreous fluid of patients with proliferative diabetic retinopathy. Life Sci 64: 2307-2315. doi:10.1016/S0024-3205(99)00184-8. PubMed: 10374894. 10374894

[10] Vandooren J, Geurts N, Martens E, Van den Steen PE, Opdenakker G (2013) Zymography methods for visualizing hydrolytic enzymes. Nat Methods 10:211: 220 PubMed: 23443633. 10.1038/nmeth.2371 23443633

[11] Paemen L, Martens E, Masure S, Opdenakker G (1995) Monoclonal antibodies specific for natural human neutrophil gelatinase B used for affinity purification, quantitation by two-site RLISA and inhibition of enztmatic activity. Eur J Biochem 234: 759-765. doi:10.1111/j.1432-1033.1995.759_a.x. PubMed: 8575432. 8575432

[12] Pulukuri SM, Rao JS (2008) Matrix metalloproteinase-1 promotes prostate tumor growth and metastasis. Int J Oncol 32: 757-765. PubMed: 18360703. 18360703 PMC2292413

[13] Goerge T, Barg A, Schnaeker EM, Poppelmann B, Shpacovitch V et al. (2006) Tumor-derived matrix metalloproteinase-1 targets endothelial proteinase-activated receptor 1 promoting endothelial cell activation. Cancer Res 66: 7766-7774. doi:10.1158/0008-5472.CAN-05-3897. PubMed: 16885380. 16885380

[14] Blackburn JS, Rhodes CH, Coon Cl, Brinckerhoff CE (2007). RNA interference inhibition of matrix metalloproteinase-1 prevents melanoma metastasis by reducing tumor collagenase activity and angiogenesis. Cancer Res 67:10849-10858

[15] Eck SM, Hoopes PJ, Petrella BL, Coon Cl, Brinckerhoff CE (2009). Matrix metalloproteinase-1 promotes breast cancer angiogenesis and osteolysis in a novel in vivo model. Breast Cancer Res Treat 116:79-90 10.1007/s10549-008-0085-3 PMC3772530 18597171

[16] Blackburn JS, Brinckerhoff CE (2008) Matrix metalloproteinase-1 and thrombin differentially activate gene expression in endothelial cells via PAR-1 and promote angiogenesis. Am J Pathol 173: 1736-1746. doi:10.2353/ajpath.2008.080512. PubMed: 18988801. 18988801 PMC2626385

[17] Pufe T, Harde V, Petersen W, Goldring MB, Tillmann B et al. (2004) Vascular endothelial growth factor (VEGF) induces matrix metalloproteinase expression in immortalized chondrocytes. J Pathol 202: 367-374. doi:10.1002/path.1527. PubMed: 14991903. 14991903

[18] Wang H, Keiser JA (1998) Vascular endothelial growth factor upregulates the expression of matrix metalloproteinases in vascular smooth muscle cells: role of flt-1. Circ Res 83: 832-840. doi:10.1161/01.RES.83.8.832. PubMed: 9776730. 9776730

[19] Steenport M, Khan KM, Du B, Barnhard SE, Dannenberg AJ et al. (2009) Matrix metalloproteinase (MMP)-1 and MMP-3 induce macrophage MMP-9: evidence for the role of TNF-alpha and cyclooxygenase-2. J Immunol 183: 8119-8127. doi:10.4049/jimmunol.0901925. PubMed: 19923455. 19923455 PMC3621723

[20] Sier CF, Hawinkels LJ, Zijlmans HJ, Zuidwijk K, de Jonge-Muller ES et al. (2008) Endothelium specific matrilysin (MMP-7) expression in human cancers. Matrix Biol 27: 267-271. PubMed: 18023162. 18023162 10.1016/j.matbio.2007.10.006

[21] Huo N, Ichikawa Y, Kamiyama M, Ishikawa T, Hamaguchi Y et al. (2002) MMP-7 (matrilysin) accelerated growth of human umbilical vein endothelial cells. Cancer Lett 177: 95-100. doi:10.1016/S0304-3835(01)00772-8. PubMed: 11809536. 11809536

[22] Ito TK, Ishii G, Saito S, Yano K, Hoshino A et al. (2009) Degradation of soluble VEGF receptor-1 by MMP-7 allows VEGF access to endothelial cells. Blood 113: 2363-2369. doi:10.1182/blood-2008-08-172742. PubMed: 18974372. 18974372

[23] Vandooren J, Van den Steen PE, Opdenakker G (2013) Biochemistry and molecular biology of gelatinase B or matrix metalloproteinase-9 (MMP-9): the next decade. Crit Rev Biochem Mol Biol 48: 222-272. doi:10.3109/10409238.2013.770819. PubMed: 23547785. 23547785

[24] Shibuya M (2006) Differential roles of vascular endothelial growth factor receptor-1 and receptor-2 in angiogenesis. J Biochem Mol Biol 39: 469-478. doi:10.5483/BMBRep.2006.39.5.469. PubMed: 17002866. 17002866

[25] Alexandrakis MG, Sfiridaki A, Miyakis S, Pappa C, Kandika E et al. (2007) Relationship between serum levels of vascular endothelial growth factor, hepatocyte growth factor and matrix metalloproteinase-9 with biochemical markers of bone disease in multiple myeloma. Clin Chim Acta, 379: 379-2:31-35 PubMed: 17234170. 10.1016/j.cca.2006.11.024 17234170

[26] Zaman K, Driscoll R, Hahn D, Werffeli P, Goodman SL et al. (2006) Monitoring multiple angiogenesis-related molecules in the blood of cancer patients shows a correlation between VEGF-A and MMP-9 levels before treatment and divergent changes after surgical vs. conservative therapy. Int J Cancer 118: 755-764. doi:10.1002/ijc.21408. PubMed: 16114015. 16114015

[27] Kim KS, Choi HM, Lee YA, Choi IA, Lee SH et al. (2011) Expression levels and association of gelatinases MMP-2 and MMP-9 and collagenases MMP-1 and MMP-13 with VEGF in synovial fluid of patients with arthritis. Rheumatol Int 31: 543-547. doi:10.1007/s00296-010-1592-1. PubMed: 20665024. 20665024

[28] Hollborn M, Stathopoulos C, Steffen A, Wiedemann P, Kohen L et al. (2007) Positive feedback regulation between MMP-9 and VEGF in human RPE cells. Invest Ophthalmol Vis Sci 48: 4360-4367. doi:10.1167/iovs.06-1234. PubMed: 17724228. 17724228

[29] Ahn GO, Brown JM (2008) Matrix metalloproteinase-9 is required for tumor vasculogenesis but not for angiogenesis: role of bone marrow-derived myelomonocytic cells. Cancer Cell 13: 193-205. doi:10.1016/j.ccr.2007.11.032. PubMed: 18328424. 18328424 PMC2967441

[30] Bekes EM, Schweighofer B, Kupriyanova TA, Zajac E, Ardi VC et al. (2011) Tumor-recruited neutrophils and neuthrophil TIMP-free MMP-9 regulate coordinately the levels of tumor angiogenesis and efficiency of malignant cell intravasation. Am J Pathol 179: 1455-1470. doi:10.1016/j.ajpath.2011.05.031. PubMed: 21741942. 21741942 PMC3157227

[31] Ardi VC, Kupriyanova TA, Deryugina EI, Quigley JP (2007) Human neutrophils uniquely release TIMP-free MMP-9 to provide a potent catalytic stimulator of angiogenesis. Proc Natl Acad Sci U S A 104: 20262-20267. doi:10.1073/pnas.0706438104. PubMed: 18077379. 18077379 PMC2154419

[32] Ardi VC, Van den Steen PE, Opdenakker G, Schweighofer B, Deryugina EI et al. (2009) Neutrophil MMP-9 proenzyme, unencumbered by TIMP-1, undergoes efficient activation in vivo and catalytically induces angiogenesis via a basic fibroblast growth factor (FGF-2)/FGFR-2 pathway. J Biol Chem 284: 25854-25866. doi:10.1074/jbc.M109.033472. PubMed: 19608737. 19608737 PMC2757987

[33] Shirasawa M, Arimura N, Otsuka H, Sonoda S, Hashiguchi T et al. (2011) Intravitreous VEGF-A in eyes with massive vitreous hemorrhage. Graefes Arch Clin Exp Ophthalmol 249: 1805-1810. doi:10.1007/s00417-011-1795-5. PubMed: 21853228. 21853228

[34] Abu El-Asrar AM, Nawaz MI, Siddiquei MM, Al-Kharashi AS, Kangave D, et al. (2013). High-mobility group box-1 induces decreased brain-derived neurotrophic factor-mediated neuroprotection in the diabetic retina. Mediators Inflamm 2013:863036 Doi: 10.1155/2013/863036. PMC3671668 23766563

